# Retraction Note: CircHAS2 promotes the proliferation, migration, and invasion of gastric cancer cells by regulating PPM1E mediated by hsa-miR-944

**DOI:** 10.1038/s41419-024-06856-7

**Published:** 2024-07-03

**Authors:** Shuo Ma, Xinliang Gu, Lei Shen, Yinhao Chen, Chen Qian, Xianjuan Shen, Shaoqing Ju

**Affiliations:** 1 Department of Laboratory Medicine, Affiliated Hospital of Nantong University, Nantong, 226001 Jiangsu China; 2 Research Center of Clinical Medicine, Affiliated Hospital of Nantong University, Nantong, 226001 Jiangsu China; 3 Medical School of Nantong University, Nantong University, Nantong, 226001 Jiangsu China; 4 Department of Urology, Affiliated Hospital of Nantong University, Nantong, 226001 Jiangsu China

Retraction Note to: *Cell Death and Disease* 10.1038/s41419-021-04158-w, published online 23 September 2021

The Editors-in-Chief have retracted this article at the author’s request. After the publication of the article, the authors realized that errors were made during the storage, naming, selection, and uploading of images for Figs. 3J, 3F, 7E, and 8H. Further investigation by the Publisher found that the following image overlaps. Therefore, the Editors-in-Chief have no longer confidence in the data presented in the article.

- Partial image overlap between Figs. 3H (MKN-45, shRNA2) and 3J (MKN-45, shRNA1)
- Partial image overlap between Figs. 7E (MKN-45, OE-NC) and 7G (MKN-45, OE-PPM1E+mimics-NC)
- Partial image overlap between Figs. 7E (MKN-45, OE-PPM1E) and 7E (MKN-45, OE-PPM1E+mimics-NC)
- Image overlap between Figs. 8H (MKN-45, shNC+OE-NC) and 8H (MKN-45, shRNA2+OE-PPM1E)

Yinhao Chen, Chen Qian, Shaoqing Ju, Xinliang Gu, and Shou Ma agree to this retraction. Authors, Lei Shen and Shaoqing Ju have not responded to any correspondence from the editor/publisher about this retraction.

